# 2028 Discovery and evaluation of FOXP3 dimerization inhibitors

**DOI:** 10.1017/cts.2018.65

**Published:** 2018-11-21

**Authors:** Ravyn Thompson, Cara Coleman, Nathan G. Dolloff

**Affiliations:** Medical University of South Carolina

## Abstract

OBJECTIVES/SPECIFIC AIMS: Immuno-oncology (IO) strategies are promising new approaches for the treatment of a variety of malignancies, including multiple myeloma (MM). Regulatory T cells (Tregs), which suppress effector T cell function, are a limitation to durable IO responses. The transcription factor FOXP3 is critical for the mature Treg phenotype. FOXP3 homodimerization is required for DNA binding and transcriptional activity, and mutations mapping to the dimerization region are associated with IPEX syndrome, resulting in dysfunctional Tregs in humans. We therefore hypothesize that inhibitors of FOXP3 dimerization will repress Treg suppression and enhance the anti-MM activity of IO. METHODS/STUDY POPULATION: To discover FOXP3 dimerization inhibitors, we are modeling FOXP3 homodimerization in vitro. Currently, we are optimizing an ALPHA screen and an ELISA-based dimerization assay using recombinant full length and truncated versions of FOXP3 to discover peptidomimetics that inhibit homodimerization. Induced Tregs expanded from human PBMCs will be treated with lead biologics and functional assays will be performed. RESULTS/ANTICIPATED RESULTS: Here we demonstrate Treg suppression of T cell proliferation and IFN-γ secretion after 5 days of co-culture under basal conditions. Additionally, we developed a MM/T cell co-culture system to measure anti-MM T cell responses and show decreased anti-MM T cell activity in the presence of Tregs. We expect to exploit the assays outlined here to demonstrate defective Treg suppression when FOXP3 dimerization is inhibited. DISCUSSION/SIGNIFICANCE OF IMPACT: These studies support drug discovery efforts that will ultimately improve IO therapies for patients with MM.

